# A hypoxia biomarker does not predict benefit from giving chemotherapy with radiotherapy in the BC2001 randomised controlled trial

**DOI:** 10.1016/j.ebiom.2024.105032

**Published:** 2024-02-21

**Authors:** Tim A.D. Smith, Catharine M.L. West, Nuradh Joseph, Brian Lane, Joely Irlam-Jones, Elisabet More, Hitesh Mistry, Kimberley J. Reeves, Yee Pei Song, Mark Reardon, Peter J. Hoskin, Syed A. Hussain, Helen Denley, Emma Hall, Nuria Porta, Robert A. Huddart, Nick D. James, Ananya Choudhury

**Affiliations:** a Translational Radiobiology Group, Division of Cancer Sciences, University of Manchester, Manchester Cancer Research Centre, Christie NHS Foundation Trust, Manchester, UK; b Nuclear Futures Institute, School of Computer Science and Electronic Engineering, Bangor University, Bangor, UK; c Sri Lanka Cancer Research Group, Maharagama, Sri Lanka; d Mount Vernon Cancer Centre, Northwood, London, UK; e Department of Oncology and Metabolism, University of Sheffield, Sheffield, UK; f Pathology Centre, Shrewsbury and Telford NHS Trust, Royal Shrewsbury Hospital, Shrewsbury, UK; g Institute of Cancer Research, Clinical Trials & Statistics Unit, London, UK; h Royal Marsden NHS Trust, Department of Oncology, Downs Road, Sutton, Surrey, England, UK

**Keywords:** Bladder cancer, Radiotherapy, Hypoxia, Gene signature, 5FU/mitomycin C, Hypofractionation

## Abstract

**Background:**

BC2001 showed combining chemotherapy (5-FU + mitomycin-C) with radiotherapy improves loco-regional disease-free survival in patients with muscle-invasive bladder cancer (MIBC). We previously showed a 24-gene hypoxia-associated signature predicted benefit from hypoxia-modifying radiosensitisation in BCON and hypothesised that only patients with low hypoxia scores (HSs) would benefit from chemotherapy in BC2001. BC2001 allowed conventional (64Gy/32 fractions) or hypofractionated (55Gy/20 fractions) radiotherapy. An exploratory analysis tested an additional hypothesis that hypofractionation reduces reoxygenation and would be detrimental for patients with hypoxic tumours.

**Methods:**

RNA was extracted from pre-treatment biopsies (298 BC2001 patients), transcriptomic data generated (Affymetrix Clariom-S arrays), HSs calculated (median expression of 24-signature genes) and patients stratified as hypoxia-high or -low (cut-off: cohort median). Primary endpoint: invasive loco-regional control (ILRC); secondary overall survival.

**Findings:**

Hypoxia affected overall survival (HR = 1.30; 95% CI 0.99–1.70; p = 0.062): more uncertainty for ILRC (HR = 1.29; 95% CI 0.82–2.03; p = 0.264). Benefit from chemotherapy was similar for patients with high or low HSs, with no interaction between HS and treatment arm. High HS associated with poor ILRC following hypofractionated (n = 90, HR 1.69; 95% CI 0.99–2.89 p = 0.057) but not conventional (n = 207, HR 0.70; 95% CI 0.28–1.80, p = 0.461) radiotherapy. The finding was confirmed in an independent cohort (BCON) where hypoxia associated with a poor prognosis for patients receiving hypofractionated (n = 51; HR 14.2; 95% CI 1.7–119; p = 0.015) but not conventional (n = 24, HR 1.04; 95% CI 0.07–15.5, p = 0.978) radiotherapy.

**Interpretation:**

Tumour hypoxia status does not affect benefit from BC2001 chemotherapy. Hypoxia appears to affect fractionation sensitivity. Use of HSs to personalise treatment needs testing in a biomarker-stratified trial.

**Funding:**

10.13039/501100000289Cancer Research UK, 10.13039/501100000272NIHR, 10.13039/501100000265MRC.

**Research in context**

**Evidence before this study**

We searched Web of Science using ‘radiotherapy AND chemoradiotherapy AND biomarker OR signature OR predictive’. The BC2001 trial showed that giving concurrent chemotherapy with radiotherapy improved the outcome of patients with muscle-invasive bladder cancer (MIBC). The BCON trial showed that having hypoxia-modifying therapy with radiotherapy improved outcomes. Both concurrent treatments are standard-of-care options in the UK. We showed previously that only patients with hypoxic tumours benefited from hypoxia modification. No one has tested whether the hypoxia status of a tumour affects benefit from giving concurrent chemotherapy with radiotherapy in patients with MIBC. A recent meta-analysis by our group has demonstrated that survival outcomes for patients in the BC2001 and BCON trials were better with hypofractionated (55Gy in 20 fractions) than conventional (64Gy in 32 fractions) radiotherapy. No previous studies have investigated how the hypoxia status of a tumours affects response to different fractionation schedules.

**Added value of this study**

We show that the benefit of giving concurrent chemotherapy with radiotherapy is similar in patients with MIBC with high and low levels of tumour hypoxia. However, the magnitude of benefit in patients with hypoxic tumours was less than that seen previously when hypoxia-modifying treatment was given with radiotherapy. We also show that patients with MIBC with hypoxic tumours do not benefit from hypofractionated radiotherapy.

**Implications of all the available evidence**

These findings indicate that bladder cancer patients should undergo assessment of tumour hypoxia status. Patients with low hypoxia tumours should receive hypofractionated radiotherapy with concurrent radiosensitising chemotherapy. Patients with high hypoxia tumours should be treated with conventional radiotherapy plus hypoxia-modification. The work also underpins the need for a prospective study to determine if patients with hypoxic tumours could benefit from hypofractionation if given concomitant hypoxia modification.

## Introduction

UK and European Association of Urology guidelines recommend either cystectomy with lymph-node dissection or radiotherapy with radiosensitisation, often preceded by neoadjuvant chemotherapy, as radical treatment for patients with muscle-invasive bladder cancer (MIBC).^1^ Although overall survival rates are comparable,^2^ radiotherapy has the advantage of bladder preservation. The curability of cancers with radiotherapy is limited by factors including intrinsic radiosensitivity and hypoxia^3^, ^4^, ^5^ with hypoxia also being associated with chemoresistance.

Two randomised trials confirmed that radiotherapy with radiosensitisation is superior to radiotherapy alone. The BC2001 phase III trial showed adding concurrent 5-fluorouracil (5-FU) and mitomycin-C (MMC) to radiotherapy increased 2-year loco-regional control by 14%.^6^ The BCON phase III trial showed a 13% improvement in 3-year overall survival when hypoxia-targeting carbogen and nicotinamide (CON) were given with radiotherapy.^7^ These improvements in survival were still evident in 10-year follow up for both trials.^8^^,^^9^ Both BC2001 and BCON allowed conventional (64 Gy/32 fractions) and hypofractionated (55 Gy/20 fractions) radiotherapy schedules. A recent meta-analysis of data from these two trials showed hypofractionation was superior to conventional radiotherapy with respect to invasive loco-regional control and non-inferior with respect to survival, while being associated with similar toxicity.^10^

There are no predictive biomarkers to aid treatment selection between surgery, radiotherapy schedule or the type of radiosensitisation. However gene expression based biomarkers for hypoxia,^11^ radiosensitivity^12^ and molecular subtype^13^ have been developed with evidence of prognostic and predictive capability.

We showed previously that patients with MIBC and high 24-gene hypoxia signature scores benefit from hypoxia-targeting therapy.^10^ The main aim of this study was to test if the 24-gene-signature would identify patients who benefited from 5-FU/MMC. Secondary aims included testing a previously published 10-gene-radiosensitivity signature (RSI) and molecular subtyping signatures for independent prognostic ability. Finally, we hypothesised that patients with hypoxic tumours would benefit less from hypofractionation due to less time for reoxygenation and a third unplanned secondary aim was added to test this hypothesis.

## Methods

### Study design and participants

This biomarker clinical validation study used retrospective samples collected from patients recruited into the BC2001 trial. Patients gave written informed consent for the use of samples in future research, and a local research ethics committee (LREC 09/H1013/24) approved the use of the samples and data in the research reported here.

Both BC2001 and BCON trial findings have been published. BC2001 (NCT00024349) was an open-label phase 3 trial with a partial 2 × 2 factorial design. Patients with a diagnosis of MIBC (stages T2 to T4N0M0) were randomised 1:1 to chemotherapy or no chemotherapy (n = 360) or to reduced high dose volume radiotherapy or not (n = 219). Chemotherapy was given concomitantly with radiotherapy: fluorouracil (500 mg/m^2^ body surface area per day on days 1–5 and days 16–20) and mitomycin C (12 mg/m^2^ on day 1). Patients received either conventional (64 Gy in 32 fractions) or hypofractionated (55 Gy in 20 fractions) radiotherapy. Each contributing centre selected their fractionation schedule for use in these trials; there was no randomisation for fractionation schedule. In a substudy, 219 patients were randomised to standard whole-bladder radiation therapy or reduced high-dose volume radiation therapy that aimed to deliver full radiation dose to the tumour and 80% of maximum dose to the uninvolved bladder.

BCON randomised (1:1) patients with bladder transitional cell carcinoma (stages T1G3N0M0 [high-grade non-muscle invasive] to T4aN0M0) to receive radiotherapy alone or with hypoxia modification using carbogen (2% CO_2_ and 98% O_2_ at 15 L/min for 5 min before and during each fraction) and nicotinamide (orally at 40–60 mg/kg, 1.5–2 h before each fraction). The same fractionation regimens were used as in BC2001.

### Gene expression analysis

Pre-treatment formalin-fixed paraffin-embedded (FFPE) samples were collected for 322 of the patients recruited into BC2001 (Supplementary Figure S1). Two 10 μm sections were taken for RNA extraction and a 4 μm section for haematoxylin and eosin staining to assess tumour content. RNA was extracted using the High Pure FFPET RNA isolation kit (Catalogue number: 06650775001, Roche, Burgess Hill, UK). We measured 260nm/280 nm (mean 1.88 ± 0.07) and 260 nm/230 nm (mean 1.71 ± 0.28) ratios by nanodrop. RNA (72 ng in a 9 μl volume) was processed to generate gene expression data with the Clariom S pico HT human array (Catalogue number: 902964, Thermo Fisher Scientific, Paisley, UK) by Yourgene Health (Manchester, UK). Batches of CEL files were GC SST (Signal Space Transformation with probe Guanine Cytosine Count Correction) RNA normalised using Affymetrix Array Power Tools (https://www.thermofisher.com/uk/en/home/life-science/microarray-analysis/microarray-analysis-partners-programs/affymetrix-developers-network.html). The log_2_ summarised gene level expression values generated were batch corrected using the ComBat function from the Bioconductor package sva. Gene expression data were generated for the BCON cohort using Affymetrix Exon arrays and have been published previously.^9^

**Fig. 1:**
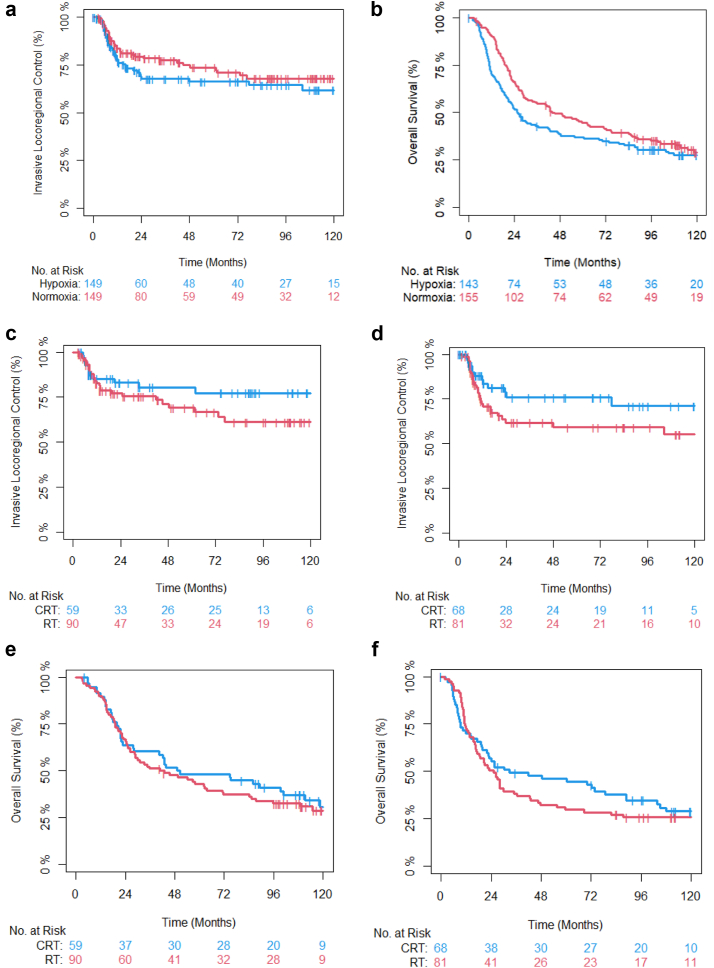
Kaplan–Meier estimates of invasive loco-regional control (ILRC) (a) and overall survival (OS) (b) of BC2001 patients (n = 298) by hypoxia status. Kaplan–Meier estimates of ILRC of BC2001 patients (n = 149) with normoxic (c) and hypoxic (d) tumours and OS of BC2001 patients with normoxic (e) and (f) hypoxic tumours stratified by treatment.

### Generation of biomarker data and centralised pathology

Hypoxia scores (HS) were calculated as the median expression of the 24 signature genes.^11^ As this is an ad-hoc exploratory analysis, hypoxia scores were analysed as both a categorical (stratified by the cohort median as in previous publications) and a continuous variable. Transcriptomic data were also used to generate radiosensitivity index (RSI) scores as previously published^12^ using the following formula: 0.0098009 ∗ AR + 0.0128283 ∗ c-Jun + 0.0254552 ∗ STAT1—0.0017589 ∗ PKC–0:0038171 ∗ RelA + 0.1070213 ∗ cABL—0.0002509 ∗ SUMO1—0.0092431 ∗ PAK2—0.0204469 ∗ HDAC1—0.0441683 ∗ IRF1. The genes were rank ordered according to their expression and coefficients for each gene multiplied by their rank. The rank of the lowest expressing gene was 1. Patient stratification with RSI was by the 25th percentile as in previous publications. The top 25% were defined as radioresistant (RSI-R), 25–75% RSI-intermediate and lowest 25% sensitive (RSI-S) as defined previously.^12^ Transcriptomic data were also used to classify samples according to consensus molecular classes^14^: luminal papillary, luminal unstable, luminal non-specified, stroma rich, basal/squamous and neuroendocrine. To improve statistical power, luminal subgroups were combined for interaction analysis. The centroid-based model was downloaded https://github.com/cit-bioinfo/BLCAsubtyping and applied as specified.^14^

The parallel 4 μm section taken from the BC2001 cohort was haematoxylin and eosin (H&E) stained and underwent centralised pathology review by an expert pathologist (H.D.). Tumour cellularity and grade were assessed. Grading was according to World Health Organisation (WHO) guidelines. Samples with <10% tumour cellularity were excluded from analysis. For analysis of RSI, samples with <50% tumour cellularity were excluded as per other publications.^15^ Grades used here were from the translational study centralised pathology review and not the original trial.

### End points

The primary endpoint in BC2001 was loco-regional disease-free survival, defined as the probability of survival free of recurrence in pelvic nodes or bladder, with data censored at the first occurrence of metastasis (if this occurred ≥30 days before loco-regional failure), a second primary tumour, or death. The primary endpoint of BCON was local relapse free survival taken as time to muscle-invasive tumour recurrence in bladder, loco-regional failure, or death. In this study, we chose an endpoint that could be defined in both BC2001 and BCON based on the information available. The primary endpoint was invasive loco-regional control (ILRC), defined as time from date of randomisation to invasive bladder recurrence or recurrence in pelvic nodes. A secondary endpoint was overall survival, defined as time from the date of randomisation to the date of death due to any cause. Patients who had not experienced an event were right-censored. We used the updated outcome data generated for comparing fractionation regimens in both trials.^10^ Endpoints were analysed up to 5 years (ILRC) or 10 years (overall survival) as in the updated outcomes papers.^8^^,^^9^

### Power analysis

For the primary end-point of ILRC, BC2001 reported a HR of 0.61 (2-year rate of 54% vs 68%) by giving concurrent chemotherapy. Access to 322 BC2001 FFPE sample were provided. The ratio of patients receiving radiotherapy alone and radiotherapy plus concurrent chemotherapy is close to 1. It is assumed that in both high-hypoxic and low-hypoxic groups, the ratio of patients receiving radiotherapy with or without concurrent chemotherapy is 1. Assuming a survival improvement from 54% to 85% by giving concurrent chemotherapy in 115 low-hypoxic patients as clinically significant, a power calculation determined that there is 81% power for a two-sided type I error of 0.01.

### Statistics

The associations between gene signatures and ILRC (primary end-point) or overall survival (secondary end-point) were assessed using Cox proportional hazards models with (multivariable) and without (univariable) inclusion of key clinical prognostic factors. The key prognostic factors were pre-specified and had been used in previous analyses of these cohorts,^9^^,^^10^ thus the same covariates were used in these analyses. Proportional hazards (PH) assumption was assessed using Schoenfeld residual plots. If the PH assumption did not hold we explored the use of flexible parametric survival models. In addition, non-linear relationship between log hazard and continuous predictors were assessed using penalised splines see^16^ with -2xlog-likelihood used as the tuning parameter to choose the optimum number of knots and their position. The penalised spline model was then compared to one without using the likelihood ratio-test likelihood and if the p-value was <0.01, the spline was retained in the model and the relationship shown graphically. For the multivariable analysis covariates were pre-specified and no covariate selection methodology was used. BC2001 and BCON cohorts were not pooled. The gene signatures were treated as both categorical and continuous covariates in the analysis. The categorical cut-off for the hypoxia signature were the median value of the cohort and for RSI the 25th percentile of the cohort (both as used in prior publications). Interaction between treatment and gene-signature was explored in BC2001 and interaction between radiotherapy fractionation schedules and gene-signatures was investigated in BC2001 and BCON. Interaction assessments were done by comparing model likelihoods with and without the interactions using the likelihood ratio-test. In addition to using the Cox proportional hazards model which assess the interaction on the multiplicative scale we also assessed the interaction using Aalens additive hazards model.^17^ The linearity assumption for Aalens additive hazards model was assessed graphically and if it did not hold the time-axis was split to account for the different slopes.

Hazard ratio for the Cox proportional hazards analyses and slopes based on the weighted linear regression to the cumulative coefficient plot for the Aalens additive hazards analysis with 95% confidence intervals for hazard ratios and p-values reported. All analyses were conducted in R v4.1.3.

### Sex verification

Self-reported and as collected in the trial case report forms.

### Ethics

Ethics approval was provided by the National Health Service (NHS) National Ethics Research Service (North-West Committee) 09/H1013/24. Patients recruited into the BC2001 trial (MREC/00/8/75) ‘consented to the donation of tissue left over from surgery and routine investigations, and to the use of excess urine samples for laboratory research that may be conducted in the future’.

### Role of the funding source

The funder of the study had no role in study design, data collection, data analysis, data interpretation, or writing of the report. All authors had full access to all data in the study and had final responsibility for the decision to submit for publication.

## Results

### Hypoxia, radiosensitivity and molecular subtype in BC2001

Gene expression data were generated for 298 BC2001 patients (Supplementary Figure S1). Failure to generate data was due to <10% tumour cellularity (Supplementary Figure S2 shows distribution of tumour cellularity) as assessed on H&E (n = 16) or poor RNA yield (n = 8). Supplementary Tables S1, S2, and S3 show the demographic and baseline disease characteristics for, respectively, the full genomic cohort, the genomic cohort stratified by hypoxia status, and the genomic cohort stratified by treatment arm. There was no evidence of a selection bias.^6^ Treatment arm, age, sex, dose/fractionation and use of neoadjuvant chemotherapy were included in multivariable analyses, but grade and stage were excluded due to low variance (Supplementary Figure S3 shows Kaplan–Meier curves for patients segregated by stage, grade and molecular subtype). Median follow-up was 119 months with an inter-quartile range of 105–135. The proportions right-censored were 28% for OS and 74% for ILRC. Reason for censoring was the event had not occurred at time of data-collection.

Supplementary Figure S4 shows the distribution of HS in 298 BC2001 patients; the median HS used as a cut-off was 6.25 (range 5.39–7.91). High hypoxia status was associated with reduced overall survival (HR = 1.30; 95% CI 0.99–1.70; p = 0.062 (Wald-test)) in multivariable analyses (Table 1). The benefit of chemotherapy was similar in patients with high versus low HS (Fig. 1c–f). Table 2 shows the results of stratified univariable and multivariable analyses indicating little evidence of an interaction between tumour hypoxia status and treatment in BC2001. Supplementary Table S4 shows the results of the interaction tests. There was no evidence of an interaction between HS and treatment arm. The tables also show findings for HS analysed as a continuous variable.

**Table 1:** Results of univariable and multivariable analyses of the BC2001 genomic cohort (n = 298).

	ILRC				OS			
	Unadjusted		Adjusted		Unadjusted		Adjusted	
	HR (95% CI)	p-value	HR (95% CI)	p-value	HR (95% CI)	p-value	HR (95% CI)	p-value
**Hypoxia– Cat.** Hypoxic v Normoxic	1.31 (0.84–2.05)	0.239	1.29 (0.82–2.03)	0.264	1.21 (0.94–1.61)	0.129	1.30 (0.99–1.70)	0.062
**Hypoxia Score– Cont.**	1.08 (0.67–1.74)	0.766	1.08 (0.67–1.75)	0.751	1.28 (0.96–1.69)	0.088	1.29 (0.97–1.71)	0.075
**RSI– Cat.**^a^ S/I v R	1.08 (0.63–1.87)	0.775	1.05 (0.60–1.84)	0.853	0.94 (0.68–1.31)	0.715	0.88 (0.63–1.23)	0.456
**RSI–Cont.**^a^	0.79 (0.17–3.59)	0.758	0.95 (0.21–4.32)	0.951	0.63 (0.25–1.59)	0.331	0.76 (0.31–1.90)	0.560
**Mol. Subtypes**^b^								
Basal/Squamous v Luminal	1.15 (0.68–1.94)	0.598	1.14 (0.67–1.93)	0.623	1.14 (0.83–1.58)	0.422	1.13 (0.81–1.56)	0.473
Stroma-Rich v Luminal	1.00 (0.50–1.99)	0.997	1.28 (0.63–2.62)	0.492	1.61 (1.13–2.31)	0.009	1.93 (1.32–2.82)	0.00069

Cat, categorical; Cont, continuous; RSI, radiosensitivity index; S/I, sensitive/intermediate; R, resistant.

Factors included in multivariable analyses were: treatment arm, age, sex, dose/fractionation and use of neoadjuvant chemotherapy.

a 284 patients were studied for RSI.

b There were too few patients in the neuroendocrine-like tumour group for statistical analysis.

**Table 2:** Stratified univariable and multivariable analysis of biomarker interaction with treatment.

	ILRC				OS			
	Unadjusted		Adjusted		Unadjusted		Adjusted	
	HR (95 CI%)	p-value	HR (95 CI%)	p-value	HR (95 CI%)	p-value	HR (95 CI%)	p-value
**Hypoxia Score—Cat.**								
CRT: H v N	1.24 (0.56–2.78)	0.596	1.23 (0.54–2.80)	0.628	1.27 (0.83–1.95)	0.269	1.33 (0.86–2.06)	0.203
RT: H v N	1.38 (0.81–2.38)	0.238	1.35 (0.79–2.33)	0.273	1.24 (0.87–1.75)	0.230	1.24 (0.87–1.75)	0.230
**Hypoxia Score—Cont.**								
CRT	1.09 (0.46–2.58)	0.851	1.09 (0.45–2.60)	0.851	1.29 (0.83–2.02)	0.263	1.33 (0.85–2.10)	0.214
RT	1.09 (0.61–1.94)	0.767	1.15 (0.63–2.09)	0.651	1.29 (0.90–1.84)	0.167	1.33 (0.91–1.93)	0.138
**RSI—Cat**.								
CRT: S/I v R	1.29 (0.52–3.20)	0.580	1.47 (0.58–3.75)	0.418	0.88 (0.53–1.45)	0.613	0.89 (0.54–1.50)	0.677
RT: S/I v R	1.08 (0.54–2.17)	0.819	0.88 (0.43–1.78)	0.715	1.02 (0.66–1.58)	0.919	0.82 (0.52–1.28)	0.373
**RSI—Cont.**								
CRT	0.65 (0.04–12.0)	0.773	0.70 (0.03–14.0)	0.814	0.64 (0.14–2.92)	0.564	0.83 (0.17–3.97)	0.815
RT	0.94 (0.16–5.39)	0.943	0.94 (0.16–5.39)	0.943	0.67 (0.21–2.15)	0.505	0.78 (0.25–2.42)	0.668
**Mol. Subtypes**								
**CRT**								
Ba./Sq. v Luminal	1.07 (0.40–2.90)	0.889	1.03 (0.38–2.84)	0.949	0.97 (0.57–1.66)	0.920	0.99 (0.58–1.71)	0.976
Str.-Rich v Luminal	1.43 (0.53–3.88)	0.485	1.58 (0.57–4.43)	0.382	1.59 (0.96–2.64)	0.070	1.65 (0.98–2.76)	0.059
**RT**								
Ba./Sq. v Luminal	1.27 (0.69–2.35)	0.438	1.22 (0.66–2.28)	0.528	1.31 (0.87–1.97)	0.030	1.24 (0.82–1.88)	0.302
Str.-Rich v Luminal	0.93 (0.33–2.63)	0.891	1.07 (0.37–3.06)	0.900	3.07 (1.12–8.46)	0.030	2.21 (1.27–3.69)	0.005

Cat, categorical; CRT, chemoradiotherapy; H, hypoxia; N, normoxic; RT, radiotherapy; Bas, basal; sq, squamous.

Factors included in multivariable analyses were: treatment arm, age, sex and use of neoadjuvant chemotherapy.

Analysis of 298 patients (284 for RSI).

**Fig. 2:**
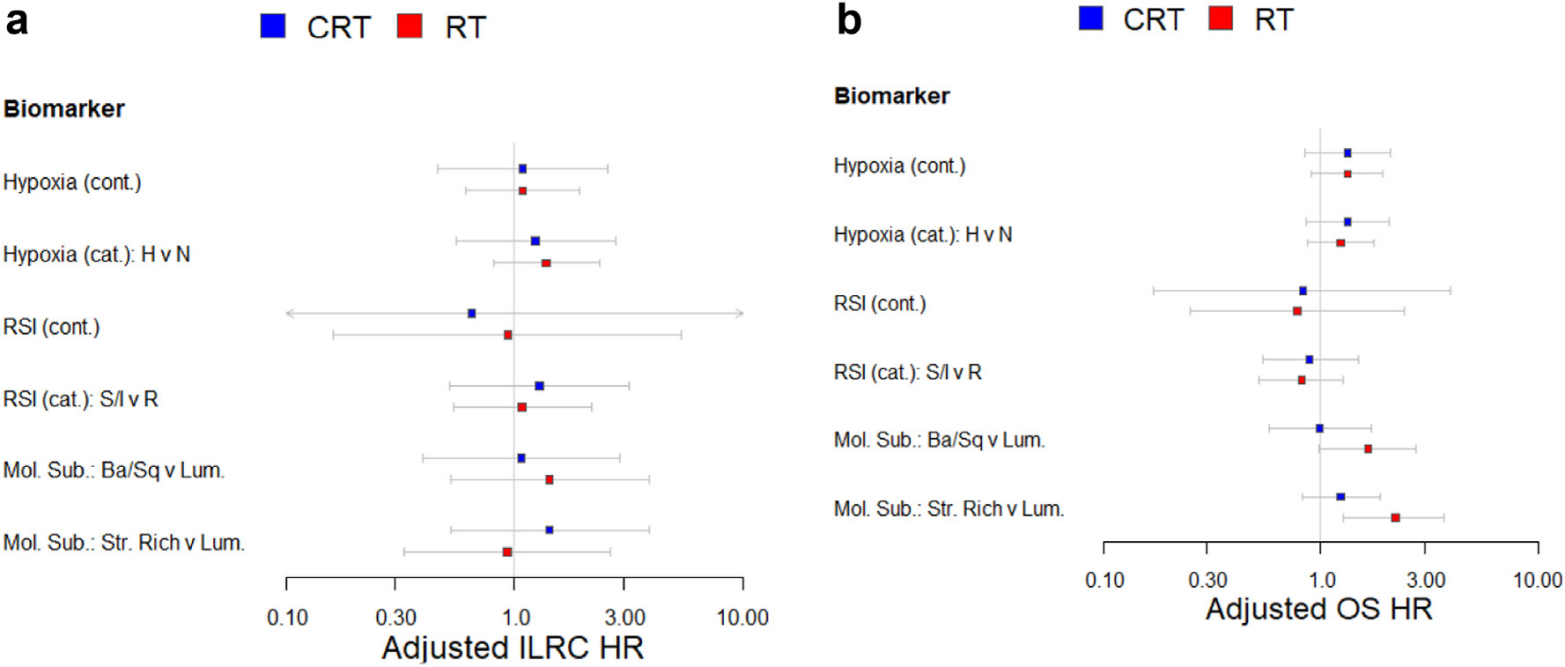
Forest plots showing adjusted ILRC (a) and overall survival (OS) (b) hazard ratios and confidence intervals for BC2001 patients stratified by tumour hypoxia score and status, radiosensitivity score and status, molecular subtype (basal squamous vs luminal and stromal rich vs luminal according to randomisation arm.

RSI was studied in 284 patients with >50% tumour cellularity. There was little evidence of differences in ILRC and OS between radiosensitive and radioresistant patients as defined by RSI score, and little evidence of an interaction with treatment in univariable or multivariable analyses (Table 2, Supplementary data Table S4, Fig. 2a and b). Molecular subtype was assessed in 298 patients. Patients with luminal tumours had a longer overall survival than patients with stromal-rich tumours, but there was little evidence of any differences in ILRC (Table 1, Fig. 2a and b), and little evidence of an interaction with treatment (Supplementary Table S4, Fig. 2a and b).

The analyses for hypoxia score were repeated using Aalens additive hazard model which gave similar directional results to the Cox proportional hazards model, see Supplementary Table S5.

### Interaction with fractionation schedule in BC2001 and BCON

As investigation of interactions with fractionation schedule were exploratory, we used the overall survival endpoint to maximise the number of events. Patient characteristics for the whole genomic BC2001 cohort who received conventional (64/32) or moderately hypofractionated (55/20) radiotherapy are shown in Supplementary Table S6. The similarities of HS, RSI and molecular subtype for the two fractionation groups are shown in Supplementary Table S7. As there was little evidence of an interaction between hypoxia and treatment in BC2001, use of chemotherapy was not expected to have an effect on the hypoxia score vs fractionation interaction analysis, and thus this was performed on the full genomic BC2001 cohort, irrespective of treatment received (chemoradiotherapy or radiotherapy only).

**Fig. 3:**
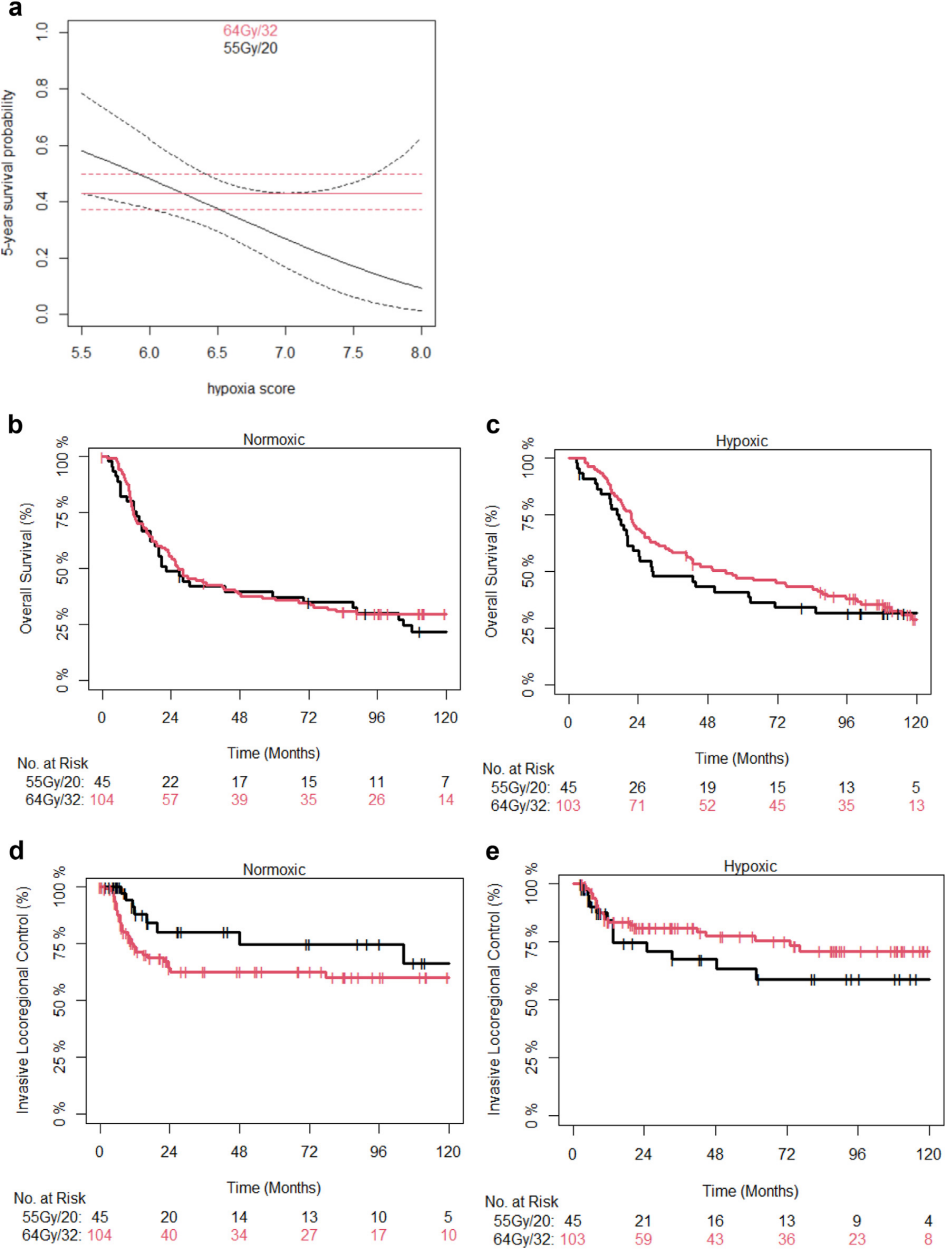
Nomogram showing the 5-year survival probability (point estimate—solid lines, 95% CI—dashed lines) as a function of hypoxia score based on whether BC2001 patients (n = 297) received conventional (64/32; red) or moderately hypofractionated (55/20; black) radiotherapy for the BC2001 patient cohort (a). The median hypoxia score is plotted as a dotted vertical line. Kaplan–Meier estimates are shown of observed ILRC and overall survival for BC2001 patients with normoxic (b and d respectively) or hypoxic (c and e respectively) tumours receiving either conventional or moderately hypofractionated radiotherapy. The point where the curves intersect in the nomogram (a) was close to the median, which was used as the hypoxia score cut-off value to generate the Kaplan-Meier plots for patients with hypoxia scores ≤6.25 (b, d) and >6.25 (c, e).

Fig. 3a shows a nomogram for the Cox proportional hazards model for the interaction of HS and 5-year survival for the whole BC2001 cohort (n = 297). The plot includes the point estimate and 95% confidence intervals for patients receiving either 64Gy/32 or 55Gy/20 for different HS values. The point where the curves intersect was close to the median, which was used to generate Kaplan-Meier plots. Fig. 3c and e shows reduced efficacy of hypofractionated radiotherapy in patients with hypoxic tumours. Table 3 shows the univariable and multivariable stratified analyses and a possible interaction of HS and fractionation regimen in BC2001.

**Table 3:** Stratified univariable and multivariable analysis of biomarker interaction with fractionation regimen.

	ILRC				OS			
	Unadjusted		Adjusted		Unadjusted		Adjusted	
	HR (95 CI%)	p-value	HR (95 CI%)	p-value	HR (95 CI%)	p-value	HR (95 CI%)	p-value
**Hypoxia Score—Cat.**								
55/20: H v N	1.73 (1.01–2.94)	0.045	1.69 (0.99–2.89)	0.057	1.31 (0.81–2.13)	0.274	1.59 (0.95–2.65)	0.076
64/32: H v N	0.61 (0.25–1.48)	0.276	0.70 (0.28–1.80)	0.461	1.20 (0.87–1.66)	0.270	1.17 (0.84–1.62)	0.358
**Hypoxia Score—Cont.**								
55/20	0.76 (0.26–2.25)	0.620	0.99 (0.35–2.75)	0.978	1.80 (1.08–2.97)	0.023	1.90 (1.15–3.16)	0.013
64/32	1.28 (0.73–2.25)	0.387	1.18 (0.37–3.76)	0.560	1.09 (0.77–1.53)	0.631	1.10 (0.78–1.55)	0.584
**RSI—Cat**.								
55/20: S/I v R	1.96 (0.66–5.86)	0.229	1.99 (0.63–6.23)	0.239	1.14 (0.67–1.94)	0.635	1.10 (0.62–1.95)	0.749
64/32: S/I v R	0.69 (0.36–1.29)	0.242	0.72 (0.38–1.36)	0.317	1.14 (0.74–1.74)	0.557	1.17 (0.77–1.79)	0.464
**RSI—Cont.**								
55/20	0.57 (0.05–6.92)	0.662	0.69 (0.07–7.14)	0.758	0.90 (0.22–3.73)	0.886	1.15 (0.29–4.53)	0.845
64/32	0.96 (0.14–6.64)	0.966	0.98 (0.14–6.71)	0.984	0.47 (0.14–1.57)	0.220	0.98 (0.14–6.71)	0.984
**Mol. Subtypes**								
**55/20**								
Ba./Sq. v Luminal	0.70 (0.25–1.96)	0.497	0.85 (0.29–2.46)	0.759	1.23 (0.69–2.19)	0.481	1.42 (0.79–2.55)	0.245
Str.-Rich v Luminal	0.99 (0.28–3.50)	0.989	1.25 (0.33–4.71)	0.744	2.20 (1.18–4.12)	0.013	2.81 (1.44–5.48)	0.003
**64/32**								
Ba./Sq. v Luminal	1.39 (0.76–2.55)	0.283	1.36 (0.73–3.52)	0.329	1.10 (0.75–1.65)	0.603	1.06 (0.71–1.58)	0.788
Str.-Rich v Luminal	1.00 (0.44–2.77)	0.997	1.48 (0.63–3.50)	0.367	1.40 (0.89–2.19)	0.141	1.78 (1.11–2.87)	0.018

H, hypoxic; N, normoxic; RSI, radiosensitivity index; S/I, sensitive/intermediate; R, resistant. Ba, basal; Sq, squamous; Str, stromal.

Factors included in multivariable analyses were: treatment arm, age, sex and use of neoadjuvant chemotherapy.

297 patients were analysed (283 for RSI) due to no information of fractionation regiment used for one patient.

**Fig. 4:**
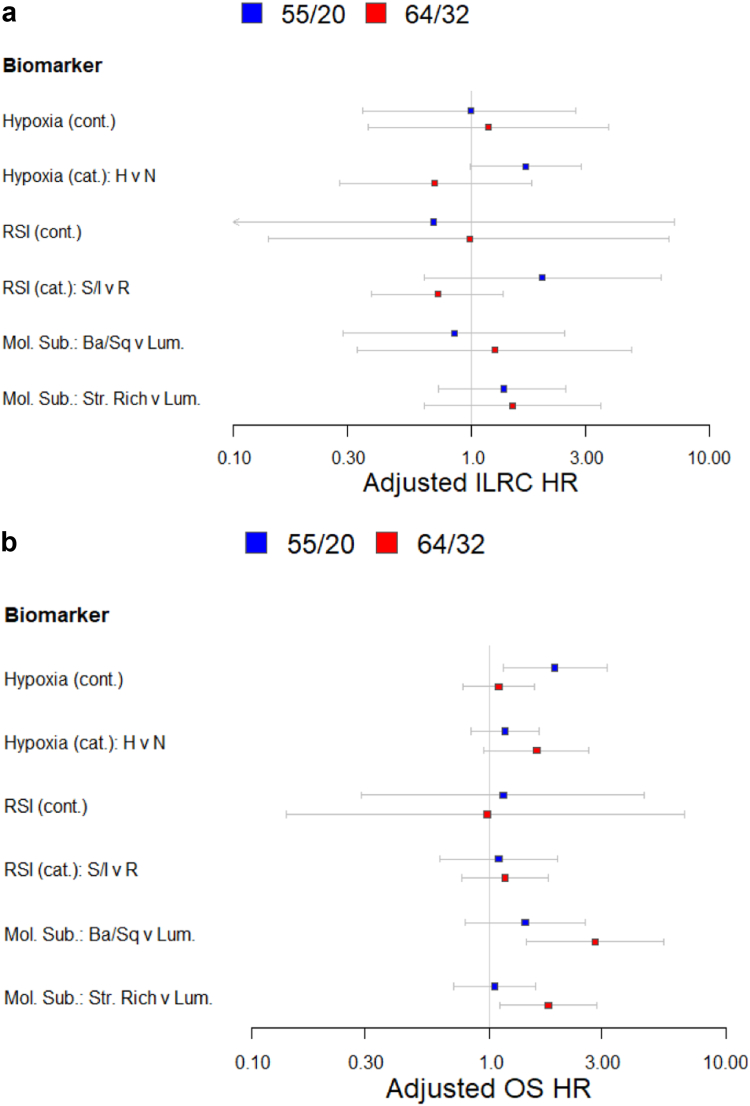
Forest plots showing adjusted ILRC (a) and overall survival (OS) (b) hazard ratios and confidence intervals for BC2001 patients stratified by tumour hypoxia score and status, radiosensitivity score and status, molecular subtype (basal squamous vs luminal and stromal rich vs luminal) according to fractionation regimen. There were too few patients in the neuroendocrine-like tumour group for statistical analysis.

Supplementary Table S8 shows the results of the interaction test. Hazard ratios and confidence plots for hypoxia score, radiosenstitivity and molecular subtypes according to fractionation regimen are shown in Fig. 4. There was evidence of an interaction between fractionation and molecular subtype for OS but not ILRC. The analyses were repeated using Aalens additive hazard model which gave similar directional results to the Cox proportional hazards model, see Supplementary Table S9.

**Fig. 5:**
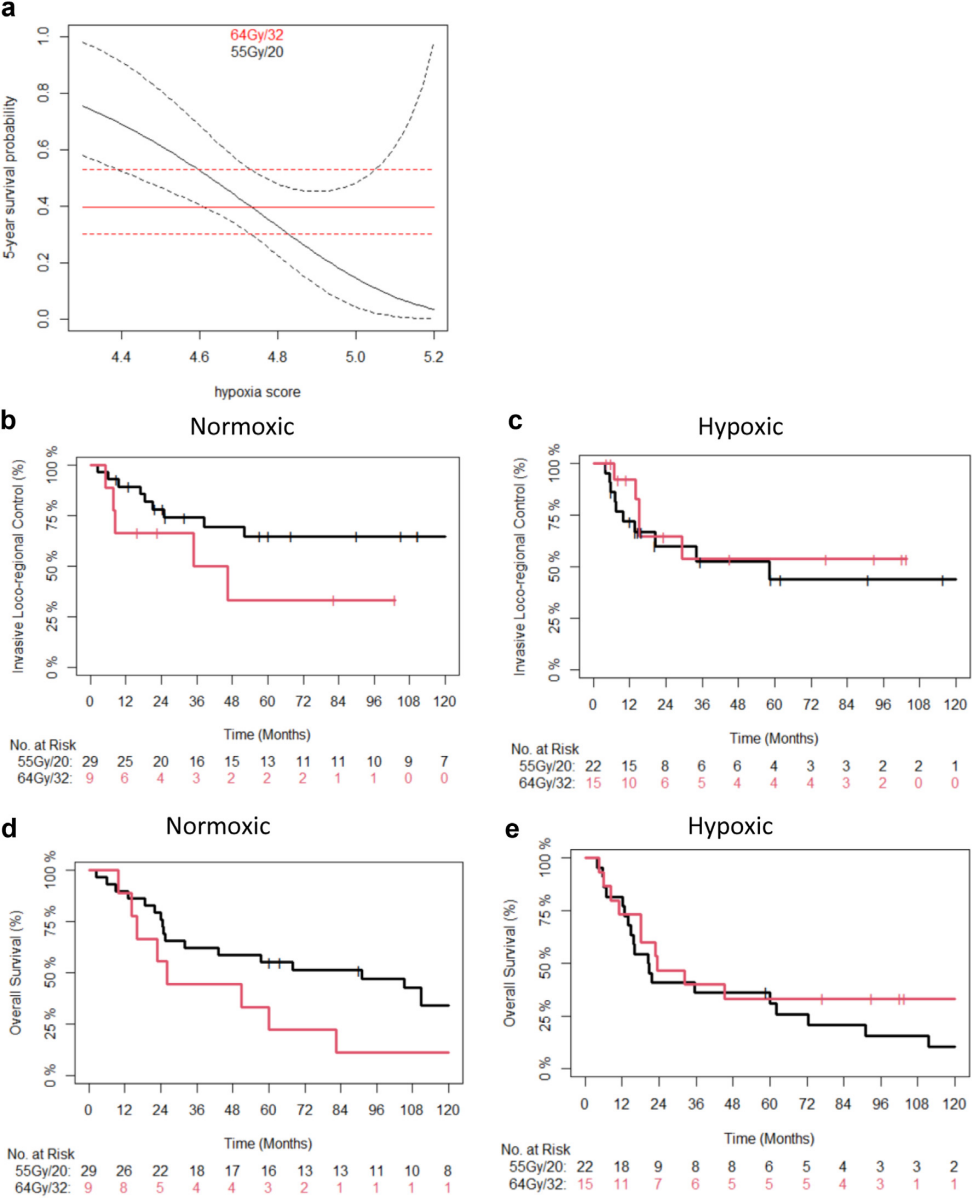
Nomogram showing the 5-year survival probability (point estimate—solid lines, 95% CI—dashed lines) as a function of hypoxia score based on whether BCON patients (n = 75) received conventional (64/32; red) or moderately hypofractionated (55/20; black) radiotherapy for the RT only arm of the BCON patient cohort (a). The median hypoxia score is plotted as a dotted vertical line. Kaplan Meier estimates are shown of observed ILRC and overall survival for BCON (RT only arm) patients with normoxic (b and d respectively) or hypoxic (c and e respectively) tumours receiving either conventional or moderately hypofractionated RT. The median hypoxia scores was used as the cut-off value to generate the KM plots for patients with hypoxia scores ≤4.7 (b, d) and >4.7 c, e).

The BCON cohort was used for validation. The analysis was undertaken in 75 patients with previously generated genomic data and who received radiotherapy alone. Similar findings to those seen in BC2001 were seen in the BCON cohort (Fig. 5). Fig. 4a shows a nomogram for the interaction of HS and that the median score provides a good cut-off point. The Supplementary Table S10 shows patients with normoxic tumours benefited from hypofractionated vs conventional radiotherapy, but there was no benefit for patients with hypoxic tumours. Hypoxia was associated with a poor prognosis for patients receiving hypofractionated radiotherapy: (n = 51; HR 14.2; 95% CI 1.7–119; p = 0.015 (Wald-test)) but not conventional radiotherapy (n = 24, HR 1.04; 95% CI 0.07–15.5, p = 0.978 (Wald-test)) (Supplementary Table S10) (Note, that the large HR seen may be due to sparse-data bias^18^). There was no apparent effect of hypoxia on fractionation sensitivity in patients receiving RT + CON (Supplementary Figure S5) suggesting hypoxia-modifying treatment abrogated the detrimental effect of hypoxia for hypofractionated radiotherapy.

## Discussion

Our study showed that the level of hypoxia in MIBC does not predict whether a patient will benefit from having chemotherapy with radiotherapy. While patients with hypoxic tumours had a poor prognosis, the survival gains achieved with chemotherapy for those with low and high signature scores were similar. Although we found RSI was not prognostic, the numbers analysed were small due to the exclusion of patients with <50% tumour material in their samples. Molecular subtype was strongly prognostic.

We hypothesised that BC2001 patients with hypoxic tumours would have a worse prognosis irrespective of treatment. There is evidence for hypoxia resistance to 5FU^19^ with mechanisms including upregulation of cell cycle inhibitors^20^ where cells are arrested in G1 (5FU is active against cells in S-phase). While hypoxia increases sensitivity to mitomycin-C in vitro,^21^ the effect is reduced in vivo due to drug cytotoxicity directed towards well oxygenated cells with low specificity for hypoxic versus oxygenated cells.^22^ This mechanism explains the absence of predictive significance with the benefit from chemotherapy being similar irrespective of hypoxia status suggesting in vitro mechanisms are not relevant at clinical doses of 5FU and mitomycin C in combination.

Sensitivity to radiotherapy is also determined by intrinsic radiosensitivity. A ten gene expression model of intrinsic radiosensitivity has been validated in multiple cancers^12^^,^^23^^,^^24^ however the current study failed to demonstrate prognostic significance in the BC2001 cohort.

Whole transcriptomic molecular studies have demonstrated that the divergent biology and prognosis of MIBC are associated with distinct molecular subtypes. To facilitate clinical translation of molecular subtyping for treatment stratification a consensus classification system with six subtypes was derived.^13^ In this study the three luminal subtypes were merged to create a luminal group with sufficient numbers for analysis. Survival of patients with stromal-rich tumours has been shown to be similar to those with luminal-papillary tumours, the subtype group with the best prognosis.^13^ In contrast in the BC2001 cohort patients with stroma-rich tumours demonstrated poor survival. This disparity may reflect the low patient numbers and the heterogeneity associated with the stroma-rich subtype.

Data on the association between radiation and molecular subtype is limited. A retrospective study of 136 patients treated with trimodality treatment showed no associated between molecular subtype and response to chemoradiation.^25^ In the BCON study the basal subtype was found to be associated with a response to hypoxia modification.^9^ This raises the possibility that molecular subtype is a potential predictive biomarker for hypoxia-targeted treatment versus chemotherapy when combining with radiation as a radiosensitiser.

There are limitations with this study. To improve statistical power luminal subtypes which do have some distinct characteristics were merged. Kaplan–Meier curves suggested a trend towards longer survival for patients with LumP subtype compared with LumU and LumNS. However, survival of patients with stromal and neuroendocrine subtypes still demonstrated poorer outcome compared with each luminal subtype. As with any clinical study, there are potential confounding factors that were not considered such as socioeconomic group and smoking status which might be expected to impact on outcome. In addition, there could be other confounding factors^26^ which may affect the choice of dose/fractionation scheme which we are unaware of, given that the choice of dose/fractionation scheme was left up to each centre's discretion in both trials. Also, our analyses of ILRC and OS were carried out at fixed time points of 5 and 10 years potentially introducing a time bias.^27^

A strength of this study is the use of robust data from phase III randomised-controlled trials with lengthy follow up, however, this could also be a limitation with a selected population rather than real world data. Further the limited patient data pool resulted in statistical underpowering that reduced the scope of some interaction assessments and in some cases the potential for multivariable analysis. The BC2001 study analysed outcome data from 360 patients to identify a 15% difference in response to radiotherapy compared with chemotherapy with 80% power. From this cohort, useable gene expression data were obtained from only 298 patients. Gene expression data were available from tumour tissue from 75 BCON patients (radiotherapy only arm) which was insufficient for multivariable analysis. RSI analysis dictates that genome expression data are only used from samples with ≥50% tumour cellularity further decreasing sample numbers to 284. Due to differences in the data available for BC2001 and BCON, we chose overall survival as an endpoint rather than disease-specific survival for validation of fraction sensitivity.

We showed previously hypofractionated radiotherapy is superior to conventional radiotherapy in unselected MIBC patients.^10^ Total effective dose was similar for the two fractionation regimens and so did not impact on the hypoxic tumour response. Using the linear-quadratic model with an α/β of 10 with a decrease in effective dose (γ) of 0.36Gy per day as demonstrated previously^28^ the biological equivalent dose for 64Gy in 32 fractions and 55Gy in 20 fractions is 71 and 70.1 Gy_10_ respectively.^10^ Shortened radiotherapy schedules provide less opportunity for reoxygenation. Our study suggests that patients with hypoxic tumours have a worse outcome with hypofractionation compared to conventional fractionation. Using linear quadratic modelling, it was demonstrated over a decade ago that tumours with hypoxic regions could be at risk of decreased tumour cell killing when treated with hypofractionated radiotherapy.^29^ This study demonstrates in two prospective clinical cohorts the detrimental effect of hypoxia when radiotherapy schedules are shortened. The BCON data analysis showed that hypoxia modification with concurrent radiotherapy improves outcome for patients with hypoxic tumours^7^^,^^9^ and that the negative impact of hypofractionation is abrogated when hypoxia modification is used. This may reflect reduced opportunity for reoxygenation when 20 fractions are used rather than 32. To the best of our knowledge no previous study has found a detrimental effect of hypoxia on reduced radiotherapy fractionation schedule. However, a tumour control probability (TCP) modelling study of patients with non-small cell lung carcinoma showed that larger tumours treated with HFRT have a lower TCP than smaller tumours, which was attributed to the higher hypoxic tumour volumes in the larger tumours.^30^

Compared with conventional radiotherapy, hypofractionation reduced the number of fractions and time from 32 over 6.5 weeks to 20 over 4 weeks. Reoxygenation, within irradiated tumours, occurs within 2–4 h^31^ with oxygen levels remaining elevated for at least 24 h post-treatment.^31^^,^^32^ The relatively poor response of hypoxic tumours to hypofractionation suggests that sustained reoxygenation to resolve chronic hypoxia^33^^,^^34^ is crucial for achieving optimum tumour control for hypoxic tumours.

With the close relationship between hypoxia and fractionation in mind,^35^, ^36^, ^37^ we hypothesised that either a delayed acceleration fractionation or temporal fractionation schedule could account for hypoxia in tumours and take advantage of progressive reoxygenation in the first few weeks of treatment.^33^ There is little clinical evidence for this effect as data sets for hypoxic tumours with different fractionation schedules are rare.

The present work has demonstrated that the bladder cancer hypoxia biomarker (1) does not predict benefit from combining 5FU/MMC with radiotherapy and (2) predicts benefit from hypofractionated radiotherapy for patients with normoxic tumours and worse outcome for patients with hypoxic tumours who do not receive hypoxia modification in two independent bladder cancer patient cohorts.

These hypothesis-confirming findings underpin the rationale for a prospective trial to identify patients with hypoxic tumours for treatment with hypoxia modification (CON) and patients with normoxic tumours to receive chemotherapy, while testing for fraction sensitivity.

## Contributors

CMLW, NDJ, RH, EM and NP designed the study; TADS, CMLW, AC and PJH prepared manuscript; HM, NP and EM statistical analysis; BL and MR transcriptomic analysis; JIJ, EM and TADS sample preparation; HD sample pathology; pathology data accessed and verified by TADS; NJ, SAH, NP, EH, NDJ, RH, and PH designed the trial. NP, EH, AC, YPS, PH and KJR managed the trial and trial data. NP, EH, RL, AC, YPS, and PH collected data. AC, NP, EH, YPS, CMLW, SAH, NDJ, RH, and PH interpreted the data. NDJ and RH were chief investigators of the BC2001 trials and PH was chief investigator of the BCON trial. The underlying data from BC2001 was accessed and verified by EH and the BCON by PH. The transcriptomic data was accessed and verified by TADS, BL, HM. All authors have read and approved the final version of the manuscript.

## Data sharing statement

All transcriptomic data from this study are deposited in the GEO repository (Geo@ncbi.nlm.nih.gov)–number GSE245953 access from 1st January 2024.

## Declaration of interests

CW: CRUK and NIHR grants, Janssen—presentation title: Hypoxia therapeutic strategy in prostate cancer, ManTRaDx—company set up to develop gene signatures to measure hypoxia—no funding received; YPS: Janssen funded meeting attendance; AC CRUK and NIHR funding, Prostate Cancer UK, UK Research and Innovation, the Urology Foundation–funding of Research programme, Janssen, Bayer, Astra Zeneca, Roche Merck, Elektra research funding; EH: Varian Medical Systems Inc—Grant received by Institution as contribution to the central trial costs of a non-commercial radiotherapy trial in prostate cancer, Accuray Inc–Grant received by Institution as contribution to the central trial costs of a non-commercial radiotherapy trial in prostate cancer, Roche products Ltd–Grant received by Institution as contribution to support central trial costs for a non-commercial trial in bladder cancer. No disclosures from the other authors.
