# Supplementary material for: A hypoxia biomarker does not predict benefit from giving chemotherapy with radiotherapy in the BC2001 randomised controlled trial
Source: eBioMedicine. 2024 Feb 21;101:105032. doi: 10.1016/j.ebiom.2024.105032 (PMC10897900; doi:10.1016/j.ebiom.2024.105032)

**SUPPLEMENTARY DATA**

| **Genomic Cohort (n=298)** | |
| --- | --- |
| **Sex – N (%)**  Female  Male | 59 (20)  239 (80) |
| **Age - years**  mean (SD) | 71.4 (7.8) |
| **RT + Radiosensitiser – N(%)** | 127 (43) |
| **Tumour Stage – N (%)**  1  2  3  4 | 1 (<1)  259 (87)  28 (9)  10 (3) |
| **Grade – N (%)**  1  2  3  Unknown | 1 (<1)  39 (13)  256 (86)  2 (1) |
| **RT Dose/Fractionation – N (%)**  55Gy/20  64Gy/32  unknown | 90 (30)  207 (69)  1 (<1) |
| **Neo-adjuvant – N (%)** | 84 (28) |
| **Hypoxia Score**  Median (IQR) | 6.29 (5.98, 6.89) |
| **RSI Score**  Median (IQR)  Not Used* - N | 0.31 (0.22, 0.43)  25 |
| **Mol. Subtypes – N (%)**  Basal/Squamous  Luminal Nonspecified  Luminal Papillary  Luminal Unstable  Neuroendocrine Like  Stroma Rich | 77 (26)  24 (8)  81 (27)  61 (20)  5 (2)  50 (17) |
| **Supplementary table 1: Patient demographics and tumour characteristics of full genomic cohort** | |

**SD – standard deviation, IQR – interquartile range
*Not used since there was less than 50% tumour material**

|  | **Normoxic**  N=149 | **Hypoxic**  N=149 |
| --- | --- | --- |
| **Sex – N (%)**  Female  Male | 34 (23)  115 (77) | 25 (17)  124 (83) |
| **Age (years)**  mean (SD) | 72.1 (7.1) | 70.7 (8.4) |
| **RT + Radiosensitiser – N(%)** | 68 (46) | 59 (40) |
| **Tumour Stage – N (%)****  1  2  3  4 | 1 (<1)  133 (89)  11 (7)  5 (3) | 0 (0)  127 (85)  17 (11)  5 (3) |
| **Grade – N (%)**  1  2  3  Unknown | 1 (<1)  13 (9)  134 (90)  1 (<1) | 0 (0)  26 (17)  122 (82)  1 (<1) |
| **RT Dose/Fractionation – N (%)**  55Gy/20  64Gy/32  unknown | 45 (30)  104 (70) | 45 (30)  103 (69)  1 (<1) |
| **Neo-adjuvant – N (%)** | 36 (24) | 48 (32) |
| **Supplementary table 2: Patient demographics and tumour characteristics in stratified into patients with hypoxic and normoxic** | | |

*There is one patient in the genomic cohort and two in the BC2001 with unknown Dose/Fractionation status; SD – standard deviation,
** Grading: genomic cohort from translational centralized pathology review (author HD)

|  | **CRT (n=127)** | **RT (n=171)** |
| --- | --- | --- |
| **Sex – N (%)**  Female  Male | 25 (20)  102 (80) | 34 (20)  137 (80) |
| **Age (years)**  Mean (SD) | 70.9 (7.5) | 71.8 (8.1) |
| **Tumour Stage – N (%)**  1  2  3  4 | 0 (0)  111 (87)  11 (9)  5 (4) | 1 (1)  148 (87)  17 (10)  5 (3) |
| **Grade – N (%)***  1  2  3  Unknown | 1 (1)  14 (11)  112 (88)  0 (0) | 0 (0)  25 (15)  144 (84)  2 (1) |
| **RT Dose/Fractionation – N (%)**  55Gy/20  64Gy/32  unknown | 39 (31)  88 (69)  0 (0) | 51 (30)  119 (69)  1 (<1) |
| **Neo-adjuvant – N (%)** | 39 (31) | 45 (26) |
| **Hypoxia Score**  Median (IQR) | 6.26 (5.98, 6.66) | 6.26 (6.00, 6.70) |
| **RSI Score**  Median (IQR)  Not Used** - N | 0.31 (0.23, 0.43)  14 | 0.30 (0.21, 0.41)  11 |
| **Mol. Subtypes – N (%)**  Basal/Squamous  Luminal Nonspecified  Luminal Papillary  Luminal Unstable  Neuroendocrine Like  Stroma Rich | 32 (25)  8 (6)  26 (20)  29 (23)  1 (1)  31 (24) | 45 (26)  16 (9)  55 (32)  32 (19)  4 (2)  19 (11) |
| **Supplementary table 3: Patient demographics and tumour characteristics of CRT and RT only genomic cohort subgroups** | | |

SD – standard deviation, IQR – interquartile range; * Grading: genomic cohort from translational centralized pathology review (author HD)
**Not used since there was less than 50% tumour material

|  | **ILRC** | | | | **OS** | | | |
| --- | --- | --- | --- | --- | --- | --- | --- | --- |
|  | **Unadjusted** | | **Adjusted** | | **Unadjusted** | | **Adjusted** | |
|  | **HR**  **(95CI%)** | **p-value** | **HR**  **(95CI%)** | **p-value** | **HR**  **(95CI%)** | **p-value** | **HR**  **(95CI%)** | **p-value** |
| Hypoxia Score – Cat.  Hypoxic v Normoxic:TRT  Hypoxic v Normoxic  TRT– RT v CRT | 1.11(0.42-2.91)  1.25 (0.56-2.78)  1.59 (0.78-3.24) | 0.839 | 0.94 (0.54-1.63)  1.16 (0.52-2.61)  1.49 (0.73-3.06) | 0.824 | 0.97(0.56-1.67)  1.27 (0.83-1.95)  1.23(0.83-1.84) | 0.999 | 0.94(0.54-1.63)  1.31(0.85-2.02)  1.23(0.82-1.85) | 0.999 |
| Hypoxia Score – Cont.  Hypoxia Score:RT  Hypoxia Score  TRT– RT v CRT | 1.00 (0.36-2.82)  1.08 (0.46-2.56)  1.63 (0.00-1181) | 0.996 | 1.14(0.42-2.30)  0.99(0.42-2.30)  0.68(0.00-492.6) | 0.798 | 0.98(0.55-1.73)  1.30 (0.83-2.04)  0.98 (0.55-1.73) | 0.862 | 1.02(0.57-1.83)  1.27(0.82-1.99)  1.02(0.03-41.4) | 0.920 |
| RSI – Cat.  S v R/I:TRT  S v R/I  TRT– RT v CRT | 0.83(0.27-2.62)  1.29 (0.77-0.52)  1.79 (0.97-3.30) | 0.757 | 0.69(0.22-2.18)  1.35 (0.54-3.37)  1.83 (0.99-3.38) | 0.524 | 1.21(0.62-2.35)  0.86(0.52-1.42)  1.07(0.77-1.49) | 0.579 | 1.06(0.54-2.08)  0.85(0.51-0.51)  1.12(0.80-1.56) | 0.860 |
| RSI – Cont.  RSI:TRT  RSI  TRT – RT v CRT | 1.45 (0.05-43.9)  0.65 (0.03-12.2)  1.48 (0.43-5.06) | 0.830 | 1.73 (0.06-53.4)  0.64 (0.03-12.35)  1.37(0.40-4.74) | 0.754 | 1.06 (0.16-7.11)  0.62 (0.14-2.82)  1.09 (0.55-2.16) | 0.950 | 1.18 (0.18-7.71)  0.69(0.15-3.12)  1.08 (0.55-2.12) | 0.865 |
| Mol. Subtypes  Ba./Sq. v Luminal:TRT  Str.-Rich v Luminal:TRT  Ba./Sq. v Luminal  Str.-Rich v Luminal  TRT– RT v CRT | 1.18(0.37-3.78)  0.64(0.15-2.72)  1.07(0.40-2.90)  1.42(0.52-3.85)  1.80(0.91-3.57) | 0.748 | 1.21 (0.37-3.93)  0.68(0.16-2.88)  1.00(0.37-2.72)  1.52(0.56-4.16)  1.74(0.88-3.45) | 0.773 | 1.34 (0.69-2.63)  1.10(0.53-2.31)  0.97 (0.57-1.66)  1.61(0.98-2.67)  1.16(0.79-1.69) | 0.756 | 1.32(0.67-2.59)  1.18(0.56-2.47)  0.95(0.55-1.63)  1.77(1.06-2.95)  1.15(0.78-1.69) | 0.763 |
| Factors included in multivariable analyses were: treatment arm, age, sex and use of neoadjuvant chemotherapy  Cat=categorical; Cont=continuous; CRT=chemoradiotherapy; H=hypoxia; N=normoxic; RT = radiotherapy; Bas=basal; sq=squamous.  Analysis of 298 patients (284 for RSI). | | | | | | | | |
| **Supplementary table 4: Results of interaction tests between hypoxia status and treatment in BC2001** | | | | | | | | |

|  | **ILRC** | | **OS** | |
| --- | --- | --- | --- | --- |
|  | **Slope (95% CI)** | **p-value** | **Slope (95% CI)** | **p-value** |
| **Hypoxia Score – Cat.**  CRT: H v N  RT: H v N | 0.002 (-0.004, 0.008)  0.008 (-0.003, 0.019) | 0.597  0.242 | 0.007 (-0.002, 0.016)  0.014 (-0.004, 0.033) | 0.261  0.235 |
| **Hypoxia Score – Cont.**  CRT  RT | 0.007 (-0.004, 0.017)  0.014 (-0.052, 0.071) | 0.261  0.235 | 0.007 (-0.002, 0.015)  0.016 (-0.004, 0.031) | 0.265  0.171 |
| **RSI – Cat**.  CRT: S/I v R  RT: S/I v R | -0.002 (-0.010, 0.008)  0.000 (-0.010, 0.011) | 0.690  0.823 | 0.004 (-0.002, 0.010)  0.002 (-0.002, 0.006) | 0.372  0.920 |
| **RSI – Cont.**  CRT  RT | -0.002 (-0.007, 0.003)  0.000 (-0.008, 0.008) | 0.775  0.940 | -0.014 (-0.034, 0.060)  -0.004 (-0.016, 0.008) | 0.550  0.490 |
| **Mol. Subtypes**  **CRT**  Ba./Sq. v Luminal  Str.-Rich v Luminal  **RT**  Ba./Sq. v Luminal  Str.-Rich v Luminal | 0.000 (-0.009, 0.008)  0.002 (-0.008, 0.009)  0.007 (-0.004, 0.018)  0.004 (-0.004, 0.012) | 0.879  0.552  0.577  0.902 | 0.003 (-0.005, 0.011)  0.018 (-0.005, 0.037)  0.015 (-0.005, 0.035)  0.035 (-0.006, 0.072) | 0.984  0.074  0.235  0.064 |
| Cat=categorical; Cont.=continuous; H=hypoxic; N=normoxic; RSI=radiosensitivity index; S/I=sensitive/intermediate; R=resistant;.Ba=basal; Sq=squamous; Str=stromal; CRT=chemoradiotherapy; RT = radiotherapy  Factors included in multivariable analyses were: treatment arm, age, sex, dose/fractionation and use of neoadjuvant chemotherapy  ^#^284 patients were studied for RSI  *There were too few patients in the neuroendocrine-like tumour group for statistical analysis.  95% CI were calculated via a non-parametric 1000 bootstrap resampling approach, with the 2.5^th^ and 97.5^th^ percentiles used. | | | | |
| **Slope Interpretation**  The sign indicates the direction of the correlation + implies increasing risk of event and – decreasing risk of event | | | | |
| **Supplementary table 5: Adjusted additive hazards analysis - Biomarker/treatment stratified analysis** | | | | |

|  | **Genomic Cohort (N=297)*** | | **BC2001 Cohort (N=456)*** | |
| --- | --- | --- | --- | --- |
|  | **64Gy/32**  (n=207) | **55Gy/20**  (n=90) | **64Gy/32**  (N=279) | **55Gy/20**  (N=177) |
| **Sex – N (%)**  Female  Male | 42 (20)  165 (80) | 17 (19)  73 (81) | 50 (18)  229 (82) | 37 (21)  140 (79) |
| **Age**  mean (SD) | 71.2 (8.3) | 71.7 (6.5) | 71.4 (8.7) | 71.6 (7.9) |
| **RT + Radiosensitiser – N(%)** | 88 (43) | 39 (43) | 111 (40) | 71 (40) |
| **Tumour Stage – N (%)**  1  2  3  4  Unknown | 1 (<1)  190 (92)  13 (6)  4 (2)  0 (0) | 0 (0)  69 (77)  15 (70)  6 (7)  0 (0) | 1 (<1)  251 (90)  20 (7)  7 (3)  0 (0) | 0 (0)  129 (73)  40 (23)  8 (5)  0 (0) |
| **Grade – N (%)***  1  2  3  Unknown | 1 (<1)  29 (14)  176 (85)  1 (<1) | 0 (0)  10 (11)  80 (89)  0 (0) | 1 (<1)  40 (14)  236 (85)  2 (<1) | 0 (0)  19 (11)  156 (89)  2 (1) |
| **Neo-adjuvant** | 49 (24) | 35 (39) | 65 (23) | 69 (39) |
| **Supplementary table 6: Patient demographics and tumour characteristics of 64/32 and 55/20 fractionation regimens in the genomic and full BC2001 cohorts** | | | | |

*There is one patient in the genomic cohort and two in the full BC2001 cohort with unknown Dose/Fractionation status; SD – standard deviation

** Grading: genomic cohort from translational centralized pathology review (author HD); BC2001 trial from original paper12

|  | **Fractionation*** | |
| --- | --- | --- |
|  | 55Gy/20  (n=90) | 64Gy/32  (n=207) |
| **Hypoxia Score**  Median (IQR) | 6.29 (5.97-6.61) | 6.29 (6.00-6.73) |
| **RSI Score**  Median (IQR)  Not Used* - N | 0.32 (0.22-0.44)  5 | 0.31 (0.22-0.40)  20 |
| **Mol. Subtypes – N (%)**  Basal/Squamous  Luminal  Neuroendocrine Like  Stroma Rich | 26 (29)  46 (51)  2 (2)  16 (18) | 51 (26)  119 (60)  3 (2)  34 (17) |
| **Supplementary table 7: Hypoxia score, RSI and molecular subtype and radiotherapy fractionation in BC2001** | | |

*There is one patient in the genomic cohort with unknown Dose/Fractionation status; SD – standard deviation, IQR – interquartile range
**Grading: BC2001 genomic cohort from translational centralized pathology review (author HD)

|  | **ILRC** | | | | **OS** | | | |
| --- | --- | --- | --- | --- | --- | --- | --- | --- |
|  | **Unadjusted** | | **Adjusted** | | **Unadjusted** | | **Adjusted** | |
|  | **HR**  **(95CI%)** | **p-value** | **HR**  **(95CI%)** | **p-value** | **HR**  **(95CI%)** | **p-value** | **HR**  **(95CI%)** | **p-value** |
| **Hypoxia Score – Cat.**  N v H:D/F  N v H  D/F – 55/20 v 64/32 | 2.91 (1.04-8.15)  0.57 (0.34-0.97)  0.53 (0.24-1.14) | 0.039 | 3.03 (1.07-8.55)  0.57 (0.34-0.98)  0.53 (0.25-1.15) | 0.034 | 0.91 (0.51-1.63)  0.83 (0.60-1.15)  1.18 (0.79-1.76) | 0.756 | 0.85 (0.47-1.52)  0.83 (0.60-1.16)  1.18 (0.79-1.76) | 0.577 |
| **Hypoxia Score – Cont**.  Hypoxia:D/F  Hypoxia  D/F – 55/20 v 64/32 | 0.75 (0.24-2.31)  1.16 (0.67-2.00)  0.75 (0.24-2.31) | 0.612 | 0.70 (0.23-2.15)  1.19 (0.68-2.07)  8.94 (0.01-11289) | 0.530 | 0.59 (0.32-1.08)  3.18 (1.10-9.21)  27.5 (0.54-1387) | 0.088 | 1.66(0.90-3.03)  1.11(0.79-1.56)  0.04(0.01-2.13) | 0.106 |
| **RSI – Cat.**  S v R/I:D/F  S v R/I  D/F – 55/20 v 64/32 | 0.39 (0.11-1.37)  1.45 (0.77-2.74)  1.19 (0.66-2.14) | 0.125 | 0.39 (0.11-1.38)  1.39 (0.74-2.62)  1.20 (0.66-2.17) | 0.129 | 1.09 (0.55-2.15)  0.89 (0.58-1.36)  1.16 (0.81-1.65) | 0.799 | 1.03 (0.52-2.04)  0.87 (0.57-1.33)  1.13 (0.79-1.61) | 0.937 |
| **RSI – Cont.**  RSI: D/F  RSI  D/F – 55/20 v 64/32 | 0.63 (0.03-14.5)  0.95 (0.14-6.57)  1.11 (0.35-3.50) | 0.773 | 0.50 (0.02-11.4)  1.23 (0.18-8.22)  1.20 (0.38-3.78) | 0.667 | 1.86 (0.29-11.9)  0.48 (0.14-1.59)  0.97 (0.49-1.91) | 0.512 | 1.59 (0.25-10.0)  0.63 (0.19-2.09)  0.97 (0.49-1.91) | 0.621 |
| **Mol. Subtypes**  Ba./Sq. v Luminal:D/F  Str.-Rich v Luminal:D/F  Ba./Sq. v Luminal  Str.-Rich v Luminal  D/F – 55/20 v 64/32 | 0.49 (0.15-1.63)  0.89 (0.20-3.98)  1.40 (0.77-2.57)  1.03 (0.45-2.33)  1.18 (0.62-2.25) | 0.489 | 0.50(0.15-1.66)  0.73(0.16-3.29)  1.39(0.75-2.57)  1.40(0.60-3.27)  1.24(0.64-2.38) | 0.508 | 1.09(0.54-3.51)  1.63(0.76-2.18)  1.12(0.75-1.66)  1.39(0.89-2.17)  1.00(0.66-1.51) | 0.454 | 1.18(0.58-2.39)  1.34(0.62-2.91)  1.07(0.72-1.60)  1.76(1.10-2.81)  0.98(0.64-1.49) | 0.737 |
| Factors included in multivariable analyses were: treatment arm, age, sex and use of neoadjuvant chemotherapy | | | | | | | | |
| **Supplementary table 8: Univariable and multivariable analysis of biomarker interaction with fractionation regimen** | | | | | | | | |

| **Adjusted Additive Hazards Analysis**  **Biomarker/Fractionation Stratified Analysis** | | | | |
| --- | --- | --- | --- | --- |
|  | **ILRC** | | **OS** | |
|  | **Slope (95% CI)** | **p-value** | **Slope (95% CI)** | **p-value** |
| **Hypoxia Score – Cat.**  55/20: H v N  64/32: H v N | 0.009 (-0.002, 0.020)  -0.004 (-0.012, 0.004) | 0.061  0.635 | 0.011 (-0.003, 0.025)  0.011 (-0.005, 0.027) | 0.118  0.252 |
| **Hypoxia Score – Cont.**  55/20  64/32 | 0.000 (-0.008, 0.007)  0.004 (-0.003, 0.011) | 0.855  0.618 | 0.020 (-0.001, 0.043)  0.007 (-0.010, 0.024) | 0.052  0.427 |
| **RSI – Cat**.  55/20: S/I v R  64/32: S/I v R | 0.014 (-0.004, 0.033)  -0.006 (-0.018, 0.006) | 0.157  0.368 | 0.007 (-0.012, 0.026)  0.004 (-0.008, 0.016) | 0.617  0.340 |
| **RSI – Cont.**  55/20  64/32 | -0.003 (-0.011, 0.005)  0.002 (-0.005, 0.009) | 0.987  0.887 | 0.002 (-0.009, 0.010)  -0.014 (-0.036, 0.008) | 0.941  0.229 |
| **Mol. Subtypes**  **55/20**  Ba./Sq. v Luminal  Str.-Rich v Luminal  **64/32**  Ba./Sq. v Luminal  Str.-Rich v Luminal | -0.003 (-0.010, 0.004)  -0.002 (-0.008, 0.006)  0.005 (-0.006, 0.015)  0.008 (-0.004, 0.020) | 0.494  0.919  0.429  0.317 | 0.004 (-0.008, 0.012)  0.039 (0.030, 0.048)  0.006 (-0.006, 0.012)  0.024 (0.016, 0.032) | 0.755  0.0089  0.729  0.032 |
| Cat=categorical; Cont.=continuous; H=hypoxic; N=normoxic; RSI=radiosensitivity index; S/I=sensitive/intermediate; R=resistant.Ba=basal; Sq=squamous; Str=stromal.  Factors included in multivariable analyses were: treatment arm, age, sex and use of neoadjuvant chemotherapy  297 patients were analysed (283 for RSI) due to no information of fractionation regiment used for one patient.  95% CI were calculated via a non-parametric 1000 bootstrap resampling approach, with the 2.5^th^ and 97.5^th^ percentiles used. | | | | |
| **Slope Interpretation**  The sign indicates the direction of the correlation + implies increasing risk of event and – decreasing risk of event | | | | |
| **Supplementary table 9: Adjusted additive hazards analysis: Biomarker/fractionation stratified analysis** | | | | |

| **ILRC** | | |
| --- | --- | --- |
| Fractionation | HR (95%CI) | p-value |
| 55/20 | 4.18 (0.22-77.9) | 0.339 |
| 64/32 | 0.31 (0.51-15.7) | 0.562 |
| **OS** | | |
| Fractionation | HR (95%CI) | p-value |
| 55/20 | 14.2 (1.7-119) | 0.015 |
| 64/32 | 1.04 (0.07-15.5) | 0.978 |
| **Supplementary table 10: Hypoxia score interaction with fraction regimen in BCON cohort** | | |

Supplementary Figure 1: Consort diagram

4 no tumour

1 low RNA

Assigned to no CT n=178

Transcriptomic data
n=111

5 no tumour

3 low RNA

7 No tumour

4 Low RNA

Transcriptomic data
n=127

Transcriptomic data n=60

FFPE blocks available n=65
n=458

FFPE blocks available
n=119

FFPE blocks available
n=138

Patients included in chemotherapy (CT) randomisation n=360
n=458

Patients excluded from the chemotherapy randomisation n=98
n=458

Assigned to CT n=182

Patients participated in BC2001
n=458

Supplementary figure 2: Distribution of tumour cellularity in BC2001 patient cohort

Supplementary figure 3: Kaplan-Meier curves segregated by (A) Stage (B) Grade (C) molecular subtype

a


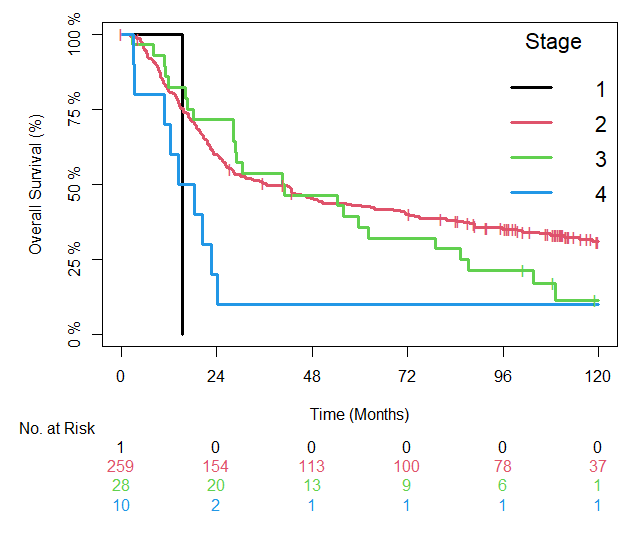


b


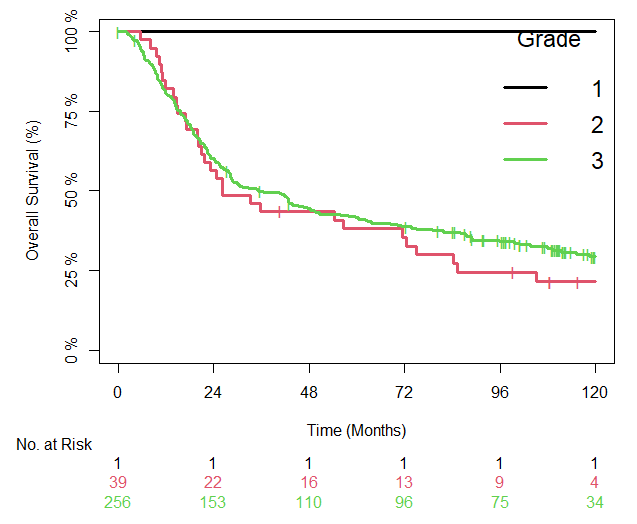


c


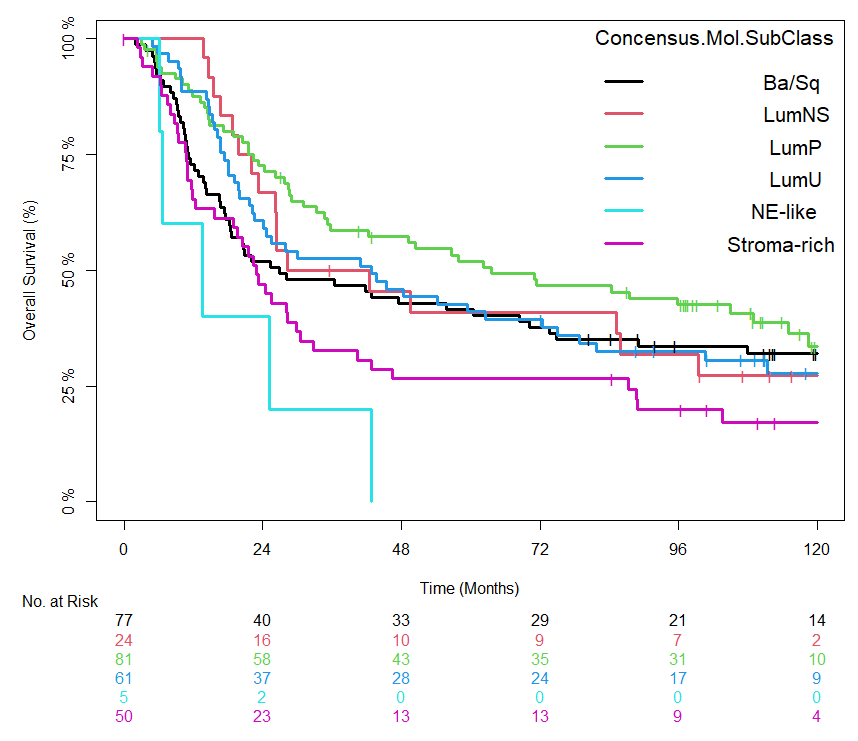


Supplementary figure 4: Distribution of hypoxia score in BC2001 patient cohort

Supplementary figure 5: Lack of interaction in RT+CON group. Kaplan-Meier estimates ILRC and OS for BCON patients who received hypoxia modification (CON) with normoxic (A and C respectively) or hypoxic (B and D respectively) tumours receiving either conventional or moderately hypofractionated radiotherapy. The point where the curves intersect in the nomogram (A) in figure 3 were used as the hypoxia score cut-off value to generate the Kaplan-Meier plots for patients with hypoxia scores ≤ 4.7 (A, C) and > 4.7 (B, D). The hypoxia score was not prognostic in the RT+CON arm therefore it was unlikely we were going to see an interaction.

a Normoxic b Hypoxic


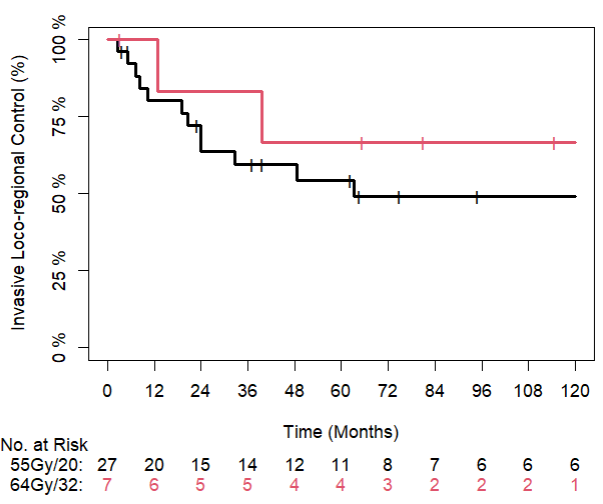

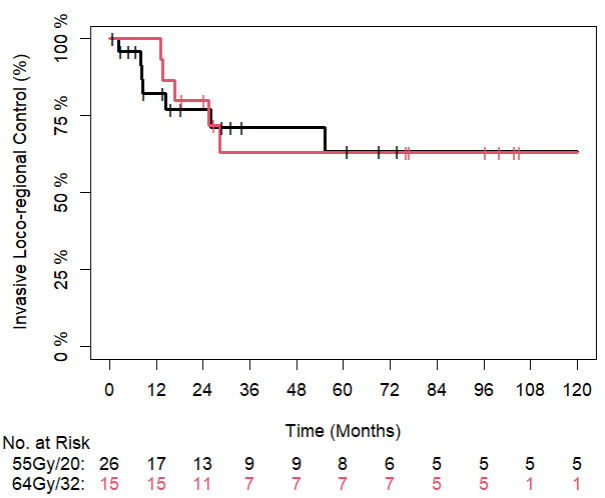


c Normoxic d Hypoxic


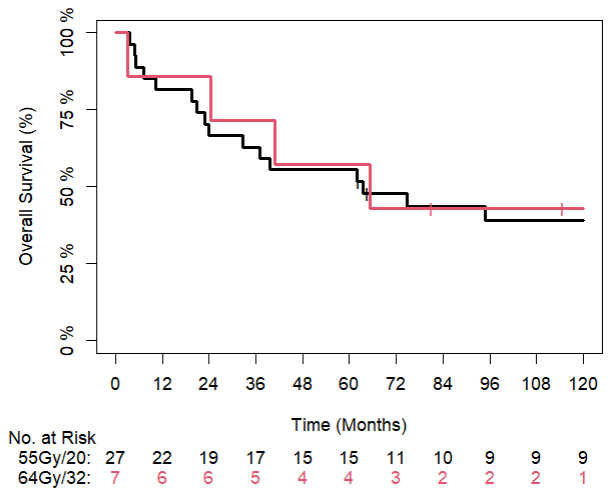

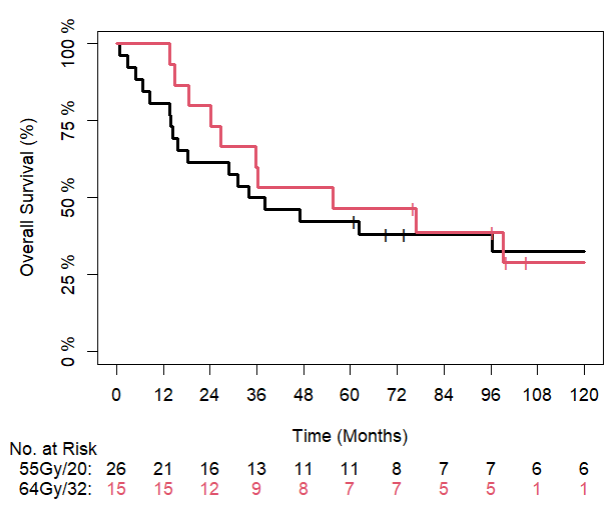

Supplement: Supplementary Tables and Figures [file mmc1.docx]
